# Coupling Mechanism of Electromagnetic Field and Thermal Stress on *Drosophila melanogaster*

**DOI:** 10.1371/journal.pone.0162675

**Published:** 2016-09-09

**Authors:** Zi-Yan Zhang, Jing Zhang, Chuan-Jun Yang, Hui-Yong Lian, Hui Yu, Xiao-Mei Huang, Peng Cai

**Affiliations:** 1 Key Laboratory of Urban Environment and Health, Institute of Urban Environment, Chinese Academy of Sciences, Xiamen, P. R. China; 2 University of the Chinese Academy of Sciences, Beijing, P. R. China; 3 Xiamen University, Xiamen, P. R. China; Universitat Zurich, SWITZERLAND

## Abstract

Temperature is an important factor in research on the biological effects of extremely low-frequency electromagnetic field (ELF-EMF), but interactions between ELF-EMF and temperature remain unknown. The effects of ELF-EMF (50 Hz, 3 mT) on the lifespan, locomotion, heat shock response (HSR), and oxidative stress (OS) of *Canton-Special* (CS) and mutant w1118 flies were investigated at 25°C and 35°C (thermal stress). Results showed that thermal stress accelerated the death rates of CS and w1118 flies, shortened their lifespan, and influenced their locomotion rhythm and activity. The upregulated expression levels of heat shock protein (*HSP*) *22*, *HSP26*, and *HSP70* indicated that HSR was enhanced. Thermal stress-induced OS response increased malondialdehyde content, enhanced superoxide dismutase activity, and decreased reactive oxygen species level. The effects of thermal stress on the death rates, lifespan, locomotion, and *HSP* gene expression of flies, especially w1118 line, were also enhanced by ELF-EMF. In conclusion, thermal stress weakened the physiological function and promoted the HSR and OS of flies. ELF-EMF aggravated damages and enhanced thermal stress-induced HSP and OS response. Therefore, thermal stress and ELF-EMF elicited a synergistic effect.

## Introduction

Extremely low-frequency electromagnetic field (ELF-EMF) poses potential health hazards and bio-effects [1]. The United Nations and the International Telecommunications Union defined the influence of EMF as a core indicator of smart sustainable urban evaluation last year [2]. Epidemiological surveys and experimental research have indicated that ELF-EMF exposure is possibly associated with diseases, such as malignancies and cardiovascular, reproductive, and neurological disorders [3–5]. ELF-EMF unlikely elicits negative biological effects [6, 7] and plays a positive role under certain conditions [8–10]. Nevertheless, the bio-effect of ELF-EMF is controversial, and its mechanism remains unclear.

The periodic movement and collision of molecules and ions are detected in alternating electromagnetic fields [11]. As such, possible ELF-EMF-induced thermal effects should be demonstrated. Although non-thermal effects induced by long-term ELF-EMF exposure have been examined [12], thermal effects should also be considered in research on the bio-effects of ELF-EMF. For instance, ELF-EMF affects heat shock protein (HSP) accumulation in cells [13, 14]. The transcript levels of numerous heat shock genes, such as *HSP22*, *HSP26*, and *HSP70*, can be altered after an individual is subjected to long-term ELF-EMF exposure [15]. Oxidative stress (OS) is another important aspect in studies concerning the bio-effects of ELF-EMF; ELF-EMF can affect the generation of reactive oxygen species (ROS) and the activity of anti-oxidative enzymes, such as superoxide dismutase (SOD), catalase (CAT), and glutathione peroxidase [16, 17]. Therefore, HSR and OS are potential pathways involved in ELF-EMF responses; in most organisms, these pathways are also activated in response to thermal stress [18, 19]. ELF-EMF and temperature elicit interacting effects; for example, ELF-EMF can reduce cell damage at low temperatures and enhance stress response at high temperatures [9, 20]. However, the coupling effects between ELF-EMF and temperature have been rarely demonstrated, and the mechanism remains unclear.

In this study, adult flies were used to examine the effects of ELF-EMF (50 Hz, 3 mT) on lifespan, locomotion, HSR, and OS at 25°C and 35°C (thermal stress). Our results indicated that ELF-EMF aggravated the thermal stress-induced damages and reduced the thermal resistance of flies, especially w1118 flies. Therefore, a possible coupling mechanism exists between ELF-EMF and thermal stress, and they elicit a synergistic effect.

## Material and Methods

### Maintenance and treatment of flies

Wild-type *Canton-Special* (CS) and mutant w1118 flies (Core Facility of *Drosophila* Resource and Technique of SIBCB, CAS) were used. The flies were maintained on a sugar-yeast standard medium in an incubator at 25°C, 60% relative humidity (RH), and 12 h/12 h light/dark cycles; the incubator lights were turned on and off at 6 a.m. and 6 p.m. [21]. One- to two-day-old male and female flies were tested separately in all of the experiments. The following four treatment groups were prepared: 25°C (normal group); 25°C + ELF (ELF-EMF group); 35°C (thermal stress group); and 35°C + ELF (thermal and ELF-EMF co-stress group). Two stress factors were stimulated and terminated synchronously in all of the experiments. The flies were anesthetized with carbon dioxide.

### ELF-EMF and thermal stress system

The ELF-EMF generating system was improved on the basis of our previous study [15]. In this study, ELF-EMF was produced by two parallel Helmholtz coils (260 turns of copper wire; diameter = 40 cm). Alternating current was applied using a variable-frequency AC power supply. The coil was electrified at 50 Hz, 82.3 V, and 6.75 A, and a variable magnetic field of 3.0 mT was produced between the two coils. The coils were then placed in an artificial climate incubator, which was utilized to control temperature, humidity, and light cycle during the experiment. A hose was wound around the coils and then connected to a condensed circulating water bath, which rapidly removed the heat produced by the coils. A temperature probe was set in the experimental zone to monitor, modify, and strictly control its actual temperature. The control system was similar to the exposure system except Helmholtz coils (S1 Fig).

### Lifespan and locomotion analysis

One- to two-day-old male and female flies were separated under low-temperature anesthesia and loaded into glass tubes (5 mm × 7 cm; 1:1 tube-fly ratio) containing 10% sucrose/2% agar food pellet at one end and a cotton pellet at the other end. The glass tubes were then placed in *Drosophila* activity monitoring 2 (DAM2) boards (TriKinetics, Inc., Waltham, MA, USA). After the flies acclimated to the new environmental conditions overnight at room temperature (25°C), the boards were subjected to the following treatment conditions: 25°C; 25°C + ELF; 35°C; and 35°C + ELF. The activities of an individual fly were determined by using infrared beams crossing the center of each tube. The cumulative counts of the flies’ activities were recorded every 5 min.

The lifespan of flies was analyzed on the basis of the duration spanning the onset of and the completion of the last activity. Kaplan-Meier survival analysis was conducted, and cumulative survival curves were plotted. Log-rank (Mantel-Cox) test was performed to determine whether the difference among groups was significant. The average lifespan, median lethal time (calculated 50% mortality age), and maximum lifespan (calculated 90% mortality age) of female and male flies were also examined.

Activity rhythm was evaluated by counting the number of activities every 30 min from the onset of stress. Activity pattern variations were analyzed on the basis of the number of activities in the first 2 h accounted for the proportion of the total number of activities within 24 h. Each experiment was performed in three replicates, with 96 males and 96 females allotted for each condition.

### RT-PCR assay

The relative expression levels of *HSP22*, *HSP26*, and *HSP70* were determined at 25°C, 25°C+ ELF, 35°C, and 35°C + ELF stress conditions. Ten surviving male and female flies were collected from each tube after 12 h of treatment. The samples were quick-frozen in liquid nitrogen for the analysis of *HSP22*, *HSP26*, and *HSP70* expression levels. The experiment was repeated thrice. Total RNA was extracted using a TRIzol reagent (Invitrogen) and was reverse-transcribed using a PrimeScript RT Master Mix Perfect Real-Time kit (Takara). Quantitative real-time PCR was performed using a SYBR Premix Ex *Taq* II kit (Takara). All of the samples were tested in triplicate, and the CT values of the target genes were normalized to those of housekeeping gene *RP49*. Normalized data were considered to quantify the relative levels of the target genes by using 2^-ΔΔCt^ method [22]. The primers used in this study are shown in S1 Table.

### OS indicator analysis

OS indicators, including ROS, malondialdehyde (MDA), total antioxidant capacity (TAC), SOD, and CAT, were examined at 25°C, 25°C + ELF, 35°C, and 35°C + ELF. Twenty male and female flies were immediately homogenized in cold phosphate buffer saline (PBS, pH 7.4) after 12 h of treatment. The supernatant was centrifuged at 2,500 × *g* and used for subsequent assays.

ROS level was detected through DCF fluorescence [23]. The supernatant (5 μl) was loaded with 295 μl of DCFH2-DA with a final concentration of 10 μM and then incubated at 37°C for 40 min. Fluorescence intensity was monitored by using a multi-mode microplate reader (Spectrum M5) at 488 nm excitation and 525 nm emission. Results were expressed as fluorescent intensity per milligram of protein.

Corresponding assay kits (Jiancheng, Nanjing, China) were used to analyze MDA content, TAC, and SOD and CAT activities. The MDA content was detected using thiobarbituric acid method. TAC was measured on the basis of the ferric-reducing ability of plasma. SOD and CAT activities were determined using xanthine oxidation and molybdenum ammonia acid methods, respectively. The experiments were replicated thrice.

### Statistical analysis

Statistical analysis was performed using SPSS 17 and Microsoft Excel. Kaplan-Meier survival analysis was conducted. A univariate general linear model was used for between-subject effects test. Post-hoc multiple comparisons (LSD) and one-way ANOVA were performed to analyze the significant differences among groups. Data were presented as mean ± standard error of the mean (SEM).

## Results

### ELF-EMF and thermal stress shortened the lifespan of flies

**Fig 1:**
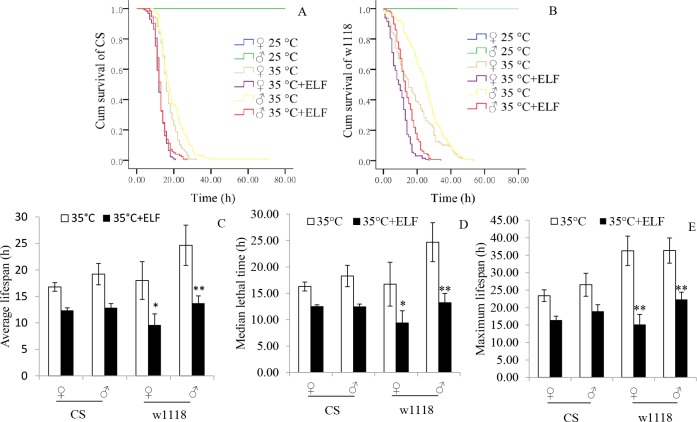
Survival functions and lifespan of flies. (A) Survival functions of CS. (B) Survival functions of w1118. (C) Average lifespan of flies. (D) Median lethal time of flies. (E) Maximum lifespan of flies. Kaplan-Meier survival analysis was conducted, and log-rank (Mantel-Cox) test was performed to analyze significant differences among groups. Time durations from the start of stress exposure to the last recorded activity were considered to analyze their lifespan. Ages of 50% and 90% mortality were used as the evaluation indexes of median lethal time and maximum lifespan, respectively. Data are presented as mean ± SEM. Asterisk (*) indicates significant difference between the male and female flies of both lines exposed to 35°C and 35°C + ELF, respectively (*P < 0.05, **P < 0.01). Flies of three replicates were used for analysis. The numbers of CS and w1118 flies were as follows: 25°C group (♀: n = 96, ♂: n = 94); 35°C group (♀: n = 128, ♂: n = 128); and 35°C + ELF group (♀: n = 124, ♂: n = 124).

The lifespan of flies was analyzed on the basis of the monitoring results of DAM2. Survival analysis results show that the death rates of the male and female CS and w1118 flies at 25°C noticeably accelerated under thermal stress. At 35°C + ELF and 35°C, the death rate of CS (♀: X^2^ = 70.93, *P < 0*.*001*; ♂: X^2^ = 71.48, *P* < 0.001; Fig 1A) and w1118 (♀: X^2^ = 50.99, *P* < 0.001; ♂: X^2^ = 99.45, *P < 0*.*001*; Fig 1B) were further accelerated by ELF-EMF exposure. Because the flies could live several weeks at 25°C, much longer than that at 35°C, so only the lifespan data of flies at 35°C were provided. The average lifespan (♀: *P = 0*.*013*; ♂: *P = 0*.*002*; Fig 1C), median lethal time (♀: *P = 0*.*038*; ♂: *P = 0*.*002*; Fig 1D), and maximum lifespan (♀: *P < 0*.*001*; ♂: *P = 0*.*002*; Fig 1E) of w1118 were significantly shortened by ELF at 35°C (S2 Table). Similar phenomena were found in the CS line, but no significant influence was observed.

### ELF-EMF and thermal stress affected fly locomotion

**Fig 2:**
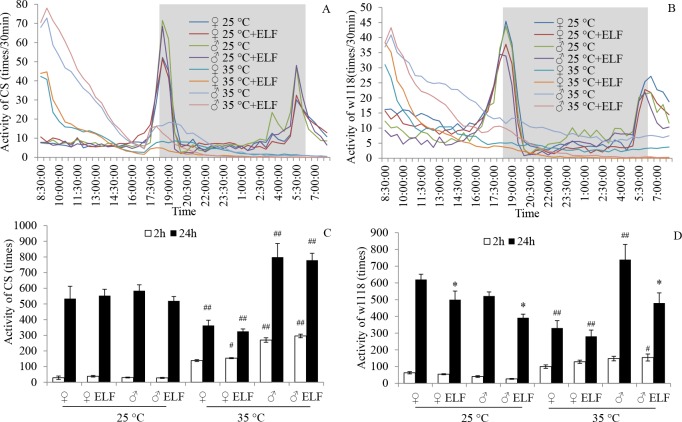
ELF-EMF and thermal stress effects on fly locomotion. Activity frequencies of flies were counted every 30 min from the beginning of stress. (A) CS locomotion rhythm in the first 24 h. (B) w1118 locomotion rhythm in the first 24 h. White and gray backgrounds in the charts represent day and night, respectively. (C) CS activity frequencies in the first 2 and 24 h. (D) w1118 activity frequencies in the first 2 and 24 h. Data are presented as mean ± SEM. Hash (^#^) indicates significant difference between groups 25°C and 35°C when other factors are the same (^#^ *P < 0*.*05*, ^##^*P < 0*.*01*). Asterisk (*) indicates significant difference between ELF-EMF and non-ELF-EMF exposure groups when other factors are the same (* *P < 0*.*05*).

The locomotion rhythm of flies was analyzed on the basis of the number of activities, which were counted every 30 min from the onset of stress. At 25°C, CS and w1118 activities for each gender followed a circadian rhythm, and two activity peaks at dawn and dusk were recorded under our experimental condition. At 35°C, the number of activities of CS and w1118 for each gender increased sharply at the beginning, declined rapidly at the following time, and maintained a low level after about 12 h (Fig 2A and 2B). Compared with that at 25°C in both CS and w1118, the number of activities in the first 24 h significantly decreased in female flies but increased in male flies at 35°C (Fig 2C and 2D). The CS flies were mainly affected by temperature (F = 28.12; *P < 0*.*001*; S3 Table), and the w1118 flies were mainly influenced by ELF-EMF (F = 11.52; *P = 0*.*002*; S3 Table). ELF-EMF exposure significantly reduced w1118 activities at 25°C (♀: *P = 0*.*045*; ♂: *P = 0*.*031*) and 35°C (♂: *P < 0*.*001*; Fig 2D). The number of activities in the first 2 h accounted for more than 30% of the total number of activities within 24 h at 35°C. By contrast, this proportion was less than 10% at 25°C.

### ELF-EMF and thermal stress increased the transcript levels of *HSP22*, *HSP26*, and *HSP70*

The transcript levels of *HSP22*, *HSP26*, and *HSP70*, which were sensitive to both ELF-EMF and thermal stress, were detected to evaluate the HSR of flies under each condition.

**Fig 3:**
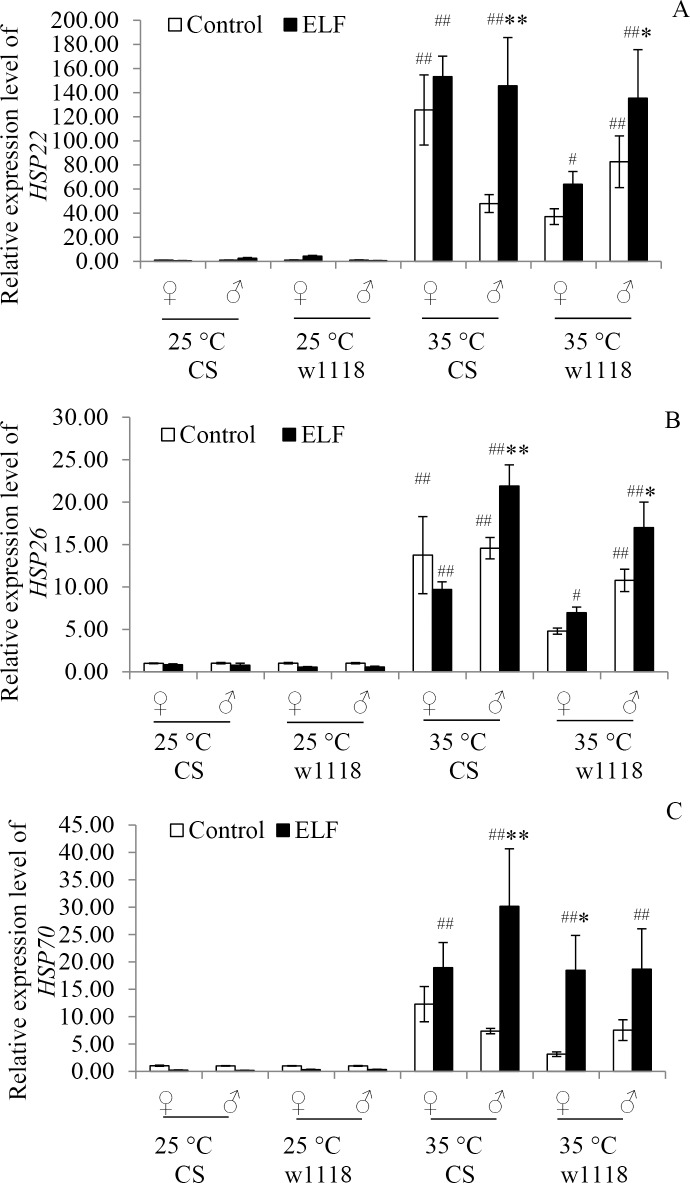
Relative transcript levels of *HSP22*, *HSP26*, and *HSP70*. Transcript levels of (A) *HSP22*, (B) *HSP26*, and (C) *HSP70* were detected 12 h after exposure to 25°C, 25°C + ELF, 35°C, and 35°C + ELF conditions. Data are presented as mean ± SEM. Hash (^#^) indicates significant difference between 25°C and 35°C groups when other factors are the same (^#^ *P < 0*.*05*, ^##^*P < 0*.*01*). Asterisk (*) indicates significant difference between ELF-EMF and non-ELF-EMF exposure groups when other factors are the same (* *P < 0*.*05*, ** *P < 0*.*01*).

*HSP22* transcript levels were mainly affected by the interaction of temperature and ELF (F = 6.58; *P = 0*.*012*; S4 Table). At 25°C, *HSP22* transcript levels were not influenced by ELF-EMF in both CS and w1118 flies. However, they were significantly upregulated at 35°C (CS♀: *P < 0*.*001*; w1118♂: *P = 0*.*002*) and at 35°C + ELF (CS♀, CS♂, and w1118♂: *P < 0*.*001*; w1118♀: *P = 0*.*044*) conditions compared with their expression levels at 25°C. Moreover, *HSP22* transcript levels at 35°C + ELF co-stress were significantly higher than those at 35°C in male flies of both CS (♀: *P = 0*.*001*) and w1118 (*P* = 0.046; Fig 3A).

*HSP26* transcript levels were mainly affected by the interaction of temperature, gender, and ELF (F = 4.57; *P = 0*.*035*; S4 Table). At 25°C, *HSP26* transcript levels were not influenced by ELF-EMF in both CS and w1118 flies. By contrast, they were significantly upregulated at 35°C (CS♀, CS♂, and w1118♂: *P < 0*.*001*) and 35°C + ELF (CS♀: *P = 0*.*001*; CS♂: *P < 0*.*001*; w1118♀: *P = 0*.*013*; w1118♂: *P < 0*.*001*) conditions compared with their expression levels at 25°C. Moreover, *HSP26* transcript levels at 35°C + ELF co-stress were significantly higher than those at 35°C in the male flies of both CS (*P = 0*.*007*) and w1118 (*P* = 0.016; Fig 3B).

*HSP70* transcript levels were mainly affected by the interaction of temperature and ELF (F = 11.39; *P = 0*.*001*; S4 Table). At 25°C, *HSP70* transcript levels were not influenced by ELF-EMF in both CS and w1118 flies. Conversely, they were significantly upregulated at 35°C and 35°C + ELF (CS♀: *P = 0*.*002*; CS♂: *P < 0*.*001*; w1118♀: *P = 0*.*003*; w1118♂: *P = 0*.*003*) conditions compared with their expression levels at 25°C. Moreover, *HSP70* transcript levels at 35°C + ELF co-stress were significantly higher than those at 35°C in male CS flies (*P = 0*.*001*) and female w1118 flies (*P* = 0.015; Fig 3C).

### ELF-EMF and thermal stress enhanced OS

OS responses were investigated to determine the underlying factors of ELF-EMF-induced effects. OS indicators, including ROS, MDA, TAC, SOD, and CAT, were detected under four conditions.

**Fig 4:**
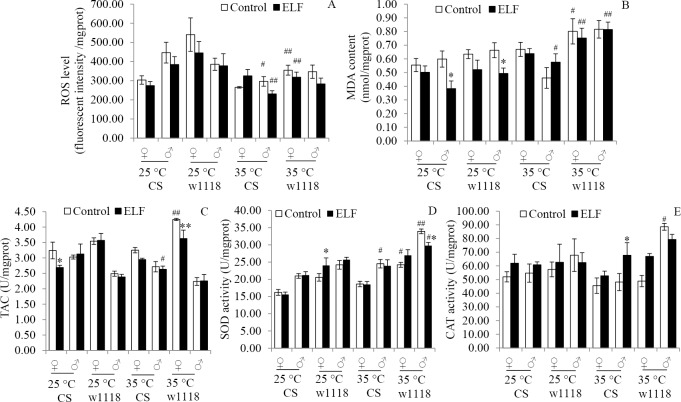
ELF-EMF and thermal stress effects on oxidative stress indicators. **(A)** ROS. (B) MDA. (C) TAC. (D) SOD. (E) CAT. OS indicators were detected after 12 h of treatments. Data are presented as mean ± SEM. Hash (^#^) indicates significant difference between 25°C and 35°C group when other factors are the same (^#^ *P < 0*.*05*, ^##^*P < 0*.*01*). Asterisk (*) indicates significant difference between ELF-EMF and non-ELF-EMF exposure groups when other factors are the same (* *P < 0*.*05*, ** *P < 0*.*01*).

ROS level was mainly influenced by temperature (F = 19.97; *P < 0*.*001*; S5 Table). Compared with those observed in the flies exposed to thermal stress at 25°C, ROS generations remarkably reduced at 35°C (CS♂: *P = 0*.*011*; w1118♀: *P = 0*.*002*) and 35°C + ELF (CS♂: *P = 0*.*009*; w1118♀: *P = 0*.*032*) (Fig 4A). At 25°C or 35°C, ELF-EMF did not significantly influence CS and w1118 flies (Fig 4A).

MDA contents were mainly influenced by the interaction of temperature and ELF-EMF (F = 6.15; *P = 0*.*014*; S5 Table). At 25°C, the MDA contents were significantly reduced by ELF-EMF in male CS (*P = 0*.*01*) and w1118 (*P = 0*.*047*) flies. Compared with that at 25°C, the MDA content increased at 35°C (w1118♀: *P = 0*.*046*) and 35°C + ELF (CS♂: *P = 0*.*021*; w1118♀: *P = 0*.*007*; w1118♂: *P < 0*.*001*) (Fig 4B).

TACs were mainly influenced by ELF-EMF (F = 5.12; *P* = *0*.*024*; S5 Table). They were significantly reduced by ELF-EMF in female CS flies (*P = 0*.*022*) at 25°C and in w1118 (*P = 0*.*01*) at 35°C. Compared with TAC at 25°C, TAC increased at 35°C (w1118♀: *P = 0*.*003*) and decreased at 35°C + ELF (CS♂: *P = 0*.*038*) (Fig 4C).

SOD activities were mainly influenced by temperature (F = 48.57, *P < 0*.*001*). They were significantly increased by ELF-EMF in female w1118 flies (*P = 0*.*034*) at 25°C and decreased in male w1118 flies (*P = 0*.*012*) at 35°C. Compared with that at 25°C, SOD increased at 35°C (CS♂: *P = 0*.*03*; w1118♀: *P = 0*.*026*; w1118♂: *P < 0*.*001*) and 35°C + ELF (CS♂: *P = 0*.*013*) (Fig 4D).

Temperature or ELF-EMF slightly influenced CAT activities (S5 Table). ELF-EMF significantly increased the CAT activities of the male flies at 35°C (*P = 0*.*04*). Compared with that at 25°C, CAT increased at 35°C (w1118 ♂: *P = 0*.*046*) (Fig 4E).

## Discussion

ELF-EMF may share similar stress-response pathways with thermal stress. As such, thermal effect should be considered in research on the bio-effects of ELF-EMF. However, coupling effects between ELF-EMF and thermal stress have been rarely investigated, and underlying mechanisms remain poorly understood. Therefore, the coupling mechanisms of ELF-EMF and thermal stress on CS and w1118 flies were investigated in this study.

The effects of temperature on flies have been widely explored. For example, the numbers of phenotypic modulations, metabolites, genes, and proteins associated with temperature damages, hardening, and tolerance have been identified [18, 24, 25]. Temperatures between 34°C and 37°C are often used as mild thermal stress conditions because acute HSR is likely induced at these temperature levels [18, 26, 27]. Likewise, our previous studies showed that the mortality of flies sharply increases, and the *HSP70* transcript level is rapidly upregulated at >35°C under short-term stress (2 h) [28]. In our present study, the physiological function of flies declined under sustained thermal stress, as supported by the acceleration of death rates, the shortening of lifespan, and the effects of locomotion rhythm and activity of flies. Under thermal stress, the metabolism of flies is enhanced in the initial stage. With prolonged exposure to stress, heat damages accumulate and metabolic rate declines because of disrupted water-salt balance, damaged cell structure, and decreased enzyme activity [29–31]. Changes in locomotion rhythm confirmed these assumptions. The activities were sharply increased in the beginning stages of the experiment and then acutely decreased in succeeding hours. Moreover, the effects of thermal stress on the death rates, lifespan, and locomotion of flies, especially w1118 line, were enhanced by ELF-EMF. These findings indicated that ELF-EMF aggravated thermal stress-induced damages, although thermal effects were also dominant at 35°C + ELF. Previous studies demonstrated similar synergistic effects; for instance, ELF-EMF enhances cellular apoptosis induced by low doses of X-ray irradiation exposure [32], increases lipid peroxidation induced by lead in mouse [33], and increases the survival rate of flies and *Escherichia coli* at low temperature [9, 34]. These findings suggested that ELF-EMF can interact with various chemical or physical factors, including temperature.

HSR and OS usually share common stress response pathways under various stress conditions [35, 36], including thermal and ELF-EMF stress. They were used to estimate the differences in stress responses at 25°C, 25°C + ELF, 35°C, and 35°C + ELF. In this study, the transcript levels of *HSP22*, *HSP26*, and *HSP70* were upregulated at 35°C possibly because the three *HSP* genes are heat-shock-induced proteins [37]. Similar variations of *HSP70* have been found in flies exposed to mild thermal stress [38]. The transcript levels of the three *HSP* genes at 35°C + ELF were higher than those at 35°C. Hence, ELF promoted *HSP* gene expression under thermal stress. ELF-EMF can strongly enhance reporter gene expression under the control of *HSP*16 and *HSP*70 promoters in mild heat shock on *Caenorhabditis elegans* [20]. Moreover, several *HSP* genes, such as *HSP16*, *HSP27*, *HSP70*, and *HSP90*, respond to ELF-EMF [14, 20, 39]. *HSP70* is closely related to self-protection mechanism [14, 40] and can be induced by ELF-EMF in flies, mice, and cells [41–43]. To the best of our knowledge, this study is the first to investigate *HSP22* and *HSP26* induced by ELF-EMF, although *HSP22* and *HSP26* have been described in other studies [15]. Similar to *HSP27* and *HSP16*, *HSP22* and *HSP26* belong to the small *HSP* family, whose expression is affected by ELF-EMF [14]. *HSP22* is mainly involved in aging, thermal, and oxidative stress pathways in flies and is necessary to adapt to stress [44, 45]. *HSP26* mainly contributes to the stress response and senility of flies [46].

Thermal stress exacerbates OS by stimulating the accumulation of harmful metabolites and affecting antioxidant enzyme activities [47]. In our study, thermal stress and ELF-EMF elicited different effects on OS indicators. Thermal stress mainly influenced ROS level, MDA content, and SOD activity. ELF-EMF mainly affected MDA content and TAC. The MDA accumulation was the combined result of ELF-EMF and thermal stress. Under thermal stress, the MDA content of the flies was increased. Thus, oxidative damages were exacerbated under ELF-EMF and thermal co-stress conditions. Decreased ROS levels may be attributed to the stimulation of OS responses, such as SOD activity enhancement. ELF-EMF combined with lead exposure also causes a remarkable increase in MDA, GSH content, and TAC of mouse brain and liver [33]. Therefore, ELF-EMF and thermal stress affect OS. However, their pathways differ, and underlying mechanisms remain unknown.

Other possible common physiological processes, such as cell membrane permeability, protein synthesis, and cell proliferation, respond to thermal and ELF-EMF stress [48]. ELF-EMF-induced responses may influence the thermal stress-induced responses of flies and vice versa under co-stress condition. Different organisms, developmental stages, and organs or tissues exhibit various sensitivities to temperature and ELF-EMF [38, 49]. For example, each part of a fly can quickly respond to temperature variations. However, the magnetic sense of flies in terms of light-dependent magneto-reception is mediated by the Cry/MagR complex [50]. Cry is mainly expressed in specific subsets of a fly’s pacemaker neurons and is in the photoreceptor cells of its compound eyes [51]. Furthermore, the intense and irregular Brownian motion of molecules and ions is dominant in thermal stress [52]. By contrast, periodical and regular Lorentz force is dominant in ELF-EMF stress [53]. When combined, these forces may achieve equilibrium. Although this phenomenon is simply a hypothesis, a mutual influence between the two forces has been widely explored in interdisciplinary physics and chemistry research [54]. The effects of ELF-EMF and thermal co-stress may have resulted from the physical and chemical dynamic balance induced by stress and be associated with different sensitivities to each factor influencing organisms.

**Fig 5:**
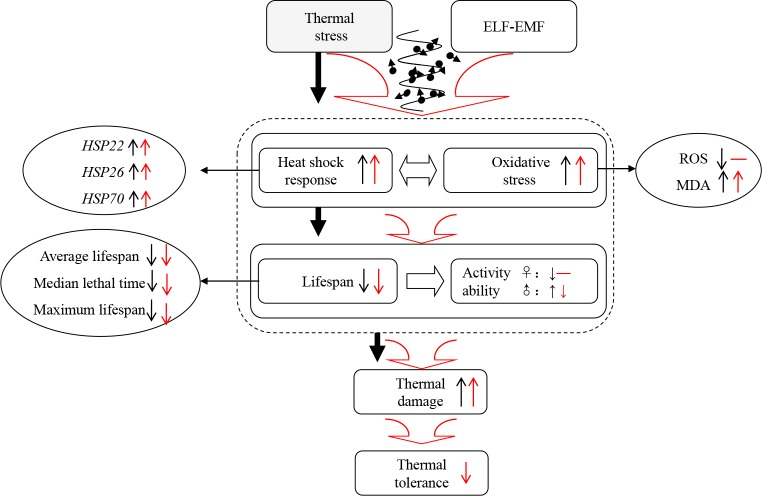
A concise summary of the study. Black arrows indicate the observations in the groups exposed to 35°C compared with those subjected to 25°C thermal stress. Red arrows correspond to the results of the groups exposed to 35°C + ELF compared with those subjected to 35°C thermal stress. Upward arrows represent an increasing trend of the corresponding factor, whereas downward arrows show a decreasing trend of the corresponding factor.

In conclusion, thermal stress weakened the physiological function and enhanced HSR and OS of CS and w1118 flies. ELF-EMF aggravated damages and enhanced thermal stress-induced HSP and OS response in flies, especially w1118 line (Fig 5). Therefore, thermal stress and ELF-EMF elicited a synergistic effect.

## Supporting Information

S1 Fig ELF-EMF and thermal stress system. ELF-EMF was produced by two parallel Helmholtz coils (260 turns of copper wire with 40 cm diameter). The coils were then placed in an artificial climate incubator, which was utilized to control temperature, humidity, and light cycle during the experiment. A hose was wound around the coils and then connected to a condensed circulating water bath, which rapidly removed the heat produced by the coils. A temperature probe was set in the experimental zone to monitor, modify and strictly control its actual temperature.
(TIF)

S1 Table Primers used in qRT-PCR. (PDF)

S2 Table Between-subject effects on average lifespan, median lethal time, and maximum lifespan. (PDF)

S3 Table Between-subject effects on activity times. (PDF)

S4 Table Between-subject effects on *HSP22*, *HSP26*, and *HPS70* transcript levels. (PDF)

S5 Table Between-subject effects on ROS, MDA, TAC, SOD, and CAT. (PDF)
