# Supplementary figures and images for: Coupling Mechanism of Electromagnetic Field and Thermal Stress on Drosophila melanogaster
Source: PLoS One. 2016 Sep 9;11(9):e0162675. doi: 10.1371/journal.pone.0162675 (PMC5017647; doi:10.1371/journal.pone.0162675)

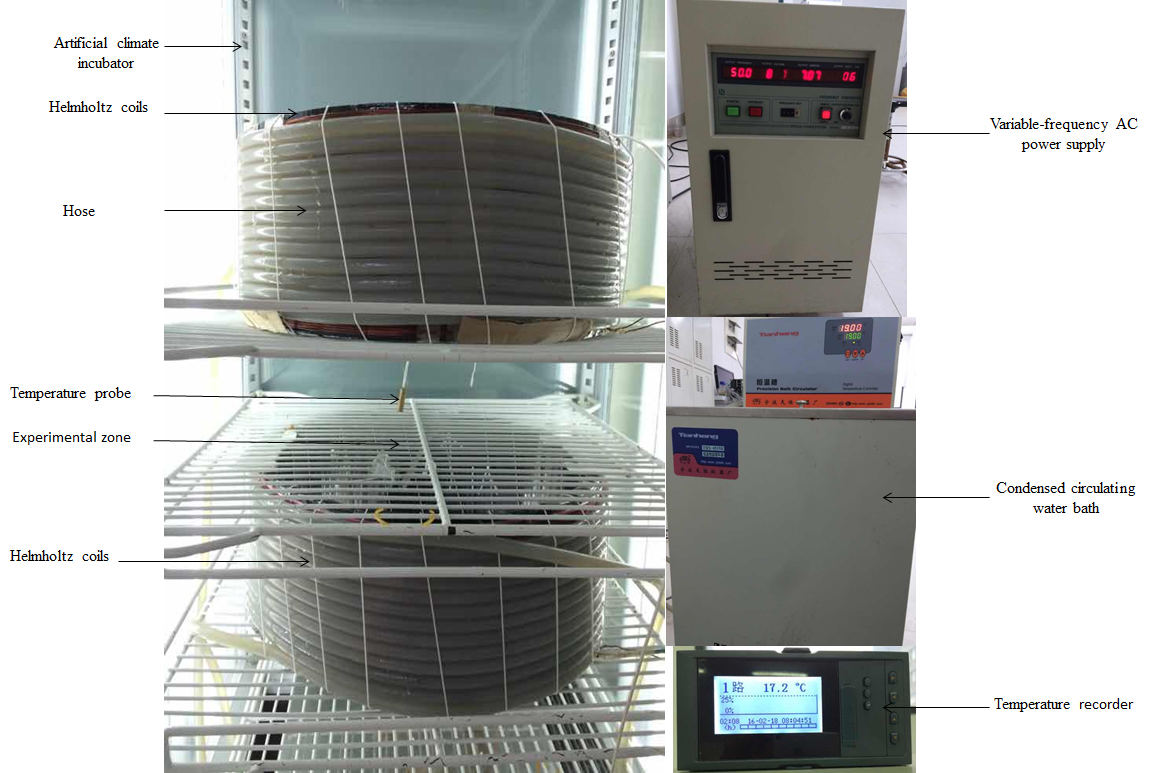

Supplement: S1 Fig — ELF-EMF was produced by two parallel Helmholtz coils (260 turns of copper wire with 40 cm diameter). The coils were then placed in an artificial climate incubator, which was utilized to control temperature, humidity, and light cycle during the experiment. A hose was wound around the coils and then connected to a condensed circulating water bath, which rapidly removed the heat produced by the coils. A temperature probe was set in the experimental zone to monitor, modify and strictly control its actual temperature. (TIF) [file pone.0162675.s001.tif]
