# Supplementary material for: Coupling Mechanism of Electromagnetic Field and Thermal Stress on Drosophila melanogaster
Source: PLoS One. 2016 Sep 9;11(9):e0162675. doi: 10.1371/journal.pone.0162675 (PMC5017647; doi:10.1371/journal.pone.0162675)
Supplement: S1 Table — (PDF) [file pone.0162675.s002.pdf]

S1 Table.

Primers used in qRT-PCR

Gene name	Forward primer(5’-3’)	Reverse primer(3’-5’)
HSP22	TGGCTACAAACTCACCCTGGA	CTGCTGCTGATTTTCCCTCC
HSP26	CGACTCCATCTTGGTCGAGG	TGTAGCCATCGGGAACCTTG
HSP70	GAACTCACACACAATGCCTGC	TCCGAGTCTGTGAAAGCCA
RP49	AGCATACAGGCCCAAGATCG	GTTGTCGATACCCTTGGGCT
