# Supplementary material for: Coupling Mechanism of Electromagnetic Field and Thermal Stress on Drosophila melanogaster
Source: PLoS One. 2016 Sep 9;11(9):e0162675. doi: 10.1371/journal.pone.0162675 (PMC5017647; doi:10.1371/journal.pone.0162675)
Supplement: S2 Table — (PDF) [file pone.0162675.s003.pdf]

S2 Table

Between-subject effects on average lifespan, median lethal time, and maximum lifespan

Dependent variable: average lifespan, median lethal time, maximum lifespan

Source	Average lifespan		Median lethal time		Maximum lifespan	
	F-Value	P-Value	F-Value	P-Value	F-Value	P-Value
Strain	0.58	0.46	0.47	0.50	9.91	0.00
Gender	4.81	0.04	4.23	0.05	2.69	0.11
ELF	23.21	0.00	18.20	0.00	39.79	0.00
Strain*Gender	1.59	0.22	2.18	0.15	0.04	0.84
Strain*ELF	1.88	0.18	1.87	0.18	6.79	0.02
Gender * ELF	0.52	0.48	0.86	0.36	0.68	0.42
Strain*Gender*ELF	0.01	0.92	0.10	0.76	0.96	0.34
