# Supplementary material for: Coupling Mechanism of Electromagnetic Field and Thermal Stress on Drosophila melanogaster
Source: PLoS One. 2016 Sep 9;11(9):e0162675. doi: 10.1371/journal.pone.0162675 (PMC5017647; doi:10.1371/journal.pone.0162675)
Supplement: S3 Table — (PDF) [file pone.0162675.s004.pdf]

S3 Table

Between-subject effects on activity times

Dependent variable: Activity

Source	Canton-s		w1118	
	F-Value	P-Value	F-Value	P-Value
Gender	59.170	0.000	6.947	0.012
Temp	28.124	0.000	0.785	0.381
ELF	0.124	0.727	11.518	0.002
Time	520.025	0.000	381.679	0.000
Gender * Temp	57.548	0.000	33.978	0.000
Gender * ELF	0.191	0.664	2.359	0.132
Gender*Time	17.864	0.000	5.506	0.024
Temp * ELF	0.024	0.878	0.000	0.995
Temp *Time	18.643	0.000	11.621	0.002
ELF *Time	0.987	0.326	12.605	0.001
Gender * Tempe * ELF	0.669	0.418	1.823	0.184
Gender * Tempe * Time	15.070	0.000	18.381	0.000
Gender * ELF * Time	0.176	0.677	1.412	0.242
Temp * ELF * Time	0.090	0.766	0.522	0.474
Gender * Tempe * ELF*Time	0.272	0.605	1.300	0.261
