# Supplementary material for: Coupling Mechanism of Electromagnetic Field and Thermal Stress on Drosophila melanogaster
Source: PLoS One. 2016 Sep 9;11(9):e0162675. doi: 10.1371/journal.pone.0162675 (PMC5017647; doi:10.1371/journal.pone.0162675)
Supplement: S4 Table — (PDF) [file pone.0162675.s005.pdf]

S4 Table

Between-subject effects on *HSP22, HSP26*, and *HPS70* transcript levels

Dependent variable: *HSP22*, *HSP26*, *HPS70*

Source	HSP22		HSP26		HSP70	
	F-Value	P-Value	F-Value	P-Value	F-Value	P-Value
Strain	3.738	0.056	8.308	0.005	1.408	0.238
Gender	0.147	0.702	16.016	0.000	0.385	0.536
Temp	99.082	0.000	163.493	0.000	40.798	0.000
ELF	7.112	0.009	2.044	0.156	9.199	0.003
Strain*Gender	6.296	0.013	0.178	0.674	0.008	0.927
Strain*Temp	3.928	0.050	7.569	0.007	1.469	0.228
Strain*ELF	0.316	0.575	0.405	0.526	0.024	0.876
Gender * Temp	0.181	0.671	16.085	0.000	0.392	0.533
Gender * ELF	1.452	0.231	4.503	0.036	0.467	0.496
Temp * ELF	6.581	0.012	3.203	0.076	11.392	0.001
Strain*Gender * Temp	7.055	0.009	0.165	0.685	0.010	0.919
Strain*Gender * ELF	0.409	0.524	0.993	0.321	1.356	0.247
Strain*Temp * ELF	0.367	0.546	0.601	0.440	0.035	0.852
Gender*Temp * ELF	1.556	0.215	4.568	0.035	0.470	0.495
Strain*Gender*Temp * ELF	0.243	0.623	1.051	0.307	1.358	0.246
