# Supplementary material for: Coupling Mechanism of Electromagnetic Field and Thermal Stress on Drosophila melanogaster
Source: PLoS One. 2016 Sep 9;11(9):e0162675. doi: 10.1371/journal.pone.0162675 (PMC5017647; doi:10.1371/journal.pone.0162675)
Supplement: S5 Table — (PDF) [file pone.0162675.s006.pdf]

S5 Table

Between-subject effects on ROS, MDA, TAC, SOD, CAT

Dependent variable: ROS, MDA, TAC, SOD, CAT

Source	ROS		MDA		TAC		SOD		CAT	
	F-Value	P-Value	F-Value	P-Value	F-Value	P-Value	F-Value	P-Value	F-Value	P-Value
Strain	10.159	0.002	22.302	0.000	1.165	0.282	118.001	0.000	11.498	0.001
Gender	0.207	0.650	1.296	0.257	87.411	0.000	74.426	0.000	9.329	0.003
Temp	19.966	0.000	24.891	0.000	0.055	0.814	48.572	0.000	0.465	0.497
ELF	3.307	0.071	4.792	0.030	5.194	0.024	0.293	0.589	3.731	0.056
Strain*Gender	7.632	0.007	3.226	0.074	55.753	0.000	0.715	0.399	2.608	0.109
Strain*Temp	0.881	0.350	5.716	0.018	1.902	0.170	3.665	0.058	3.330	0.070
Strain*ELF	0.424	0.516	0.398	0.529	0.058	0.810	1.038	0.310	1.648	0.202
Gender * Temp	0.698	0.405	0.254	0.615	11.030	0.001	3.137	0.079	4.717	0.032
Gender * ELF	0.356	0.552	0.013	0.909	4.214	0.042	3.412	0.067	1.230	0.270
Temp * ELF	0.293	0.589	6.145	0.014	0.473	0.493	2.162	0.144	0.550	0.460
Strain*Gender * Temp	9.031	0.003	1.328	0.251	0.010	0.922	1.933	0.167	0.935	0.335
Strain*Gender * ELF	1.745	0.189	0.001	0.977	0.290	0.591	4.075	0.046	3.039	0.084
Strain*Temp * ELF	0.251	0.617	0.269	0.605	0.768	0.382	1.714	0.193	0.005	0.946
Gender*Temp * ELF	1.579	0.211	3.066	0.082	0.258	0.613	1.877	0.173	0.000	0.985
Strain*Gender*Temp * ELF	0.017	0.896	0.763	0.384	3.189	0.077	0.591	0.443	1.534	0.218
